# Equine genital and ocular squamous cell carcinomas: clinical, histopathological, molecular and viral characterization with proposed histopathological classification system

**DOI:** 10.1080/01652176.2026.2648939

**Published:** 2026-03-24

**Authors:** Kevin O'Brien, Tim Mair, Hardeep S. Mudhar, Patricia Pesavento, Henry Miller, Simon L. Priestnall, Alejandro Suárez-Bonnet

**Affiliations:** a Department of Pathobiology and Population Sciences, The Royal Veterinary College, Hertfordshire, UK; b Bell Equine Veterinary Clinic, Kent, UK; c Department of Histopathology, National Specialist Ophthalmic Pathology Service (NSOPS), Royal Hallamshire Hospital, Sheffield, UK; d Department of Pathology, Microbiology and Immunology School of Veterinary Medicine, UC Davis, Davis, CA, USA

**Keywords:** Horse, papillomavirus, neoplasia, skin, squamous cell carcinoma, genital, histopathology, ocular

## Abstract

Equine squamous cell carcinomas (eSCCs) are common, and a proportion are likely induced by *Equus caballus* papillomavirus 2 (EcPV-2). Accurate prediction of clinical outcomes is challenging with no recognized prognostic criteria or consistent histopathological classification scheme for eSCC. The aims of this study were to histopathologically subtype a large case series of eSCCs (genital and ocular) and correlate them with p16 and HER-2 expression, equine papillomavirus infection status, and various clinical and histopathological parameters to predict tumour behavior and prognosis. One hundred and eighty-five samples were examined and subtyped histologically. HER-2 and p16 immunohistochemistry (IHC) and *in situ* hybridization (ISH) for the EcPV-2 E6/E7 oncogenes were performed on a subset of cases, and follow-up survival data were analyzed. The results were compared and correlated with published guidelines on the categorization of human SCC. Six histopathological subtypes of SCC, according to the WHO, were identified for the first time in horses: usual/invasive (most common), verrucous, pseudoglandular, papillary, warty, and basaloid, with different histological subtypes demonstrating prognostic significance. HER-2 and EcPV-2 statuses were not associated with prognosis in horses with SCC. p16 expression is not associated with EcPV-2 status but could be a potential prognostic factor. ISH demonstrated EcPV-2 genetic material in the majority of eSCCs, except for the papillary subtype, which includes mature, not just pre-cancerous, eSCCs. Widespread HER-2 expression in eSCCs could suggest a role for this cell receptor as a potential therapeutic target.

## 1. Introduction

Squamous cell carcinoma (SCC) is the second most common equine skin tumour (Knottenbelt et al., 2015). Equine SCC (eSCC) preferentially occurs in non-pigmented skin and mucocutaneous junctions, such as the external genitalia and eyelids of both sexes (Scott et al. 2003; van den Top et al. 2010; Armando et al. 2020). In geldings, it is the most common tumour of the penis, and it is the most common tumour of the eye and surrounding structures in horses (Davidson 1991; van den Top et al. 2008b; Iorga et al. 2020; Armando et al. 2021; Tuomisto et al. 2024). Both genital and ocular eSCCs are locally destructive, invade lymphatics and may metastasize to lymph nodes or more widely (Sykora and Brandt 2017; Sykora et al. 2017).

The etiology of eSCC is multifactorial, but high UV-radiation exposure and infection by *Equus caballus* papillomavirus-2 (EcPV-2) are the two main proposed causes (Chaux and Cubilla 2012; Sykora and Brandt 2017; Sykora et al. 2017). Papillomaviruses are species-specific, epitheliotropic, double-stranded DNA viruses (Sykora and Brandt 2017; Sykora et al. 2017). Human papilloma viruses (HPVs) are subdivided into ‘high risk’ and ‘low risk’ relating to their oncogenic potential (Muñoz et al. 2003; Iorga et al. 2020). The association between HPVs and cervical carcinoma is established, and the contribution of high-risk HPVs, such as HPV-16 and 18 (Wiener et al. 1992; Cupp et al. 1995; Frisch et al. 1999; de Villiers et al. 2004; DiMaio and Liao 2006; Muñoz et al. 2006; Pascual et al. 2007; Bogaert et al. 2012), in epithelial tumours of the penis, vagina, vulva, and anal region is well recognised (Gross and Pfister 2004; Denny and Ngan 2006; Madsen et al. 2008a, 2008b; Zhu et al. 2015). Equine lesions may initially be squamous papillomas or papillomatous plaques, with the potential to progress to squamous carcinoma *in situ*, and eventually SCC, which metastasize in 12–15% of equine cases (Scott et al. 2003; van den Top et al. 2008a, 2008b; Sykora and Brandt 2017; Sykora et al. 2017).

High-risk HPV-induced precancerous penile lesions and SCCs in people are reported to display histopathological, cytological, immunophenotypic, molecular, and immunological characteristics analogous to those of horses (Cervantes-Arias et al. 2013; Arthurs et al. 2020; Porcellato et al. 2020; Armando et al. 2021; Mecocci et al. 2021). Due to the association with EcPV-2, equine genital SCC has recently been proposed as a spontaneous large animal model for HPV-induced SCCs (van den Top et al. 2008a, 2008b; Scase et al. 2010, 2011; Knight et al. 2011; Bogaert et al. 2012; Kainzbauer et al. 2012; Sykora et al. 2012; Knight et al. 2013; Lange et al. 2013; Fischer et al. 2014; Newkirk et al. 2014; Zhu et al. 2015; Armando et al. 2021).

Several histopathological subtypes of SCC have been described in humans. Tumours associated with HPV infection possess strikingly different characteristics from those in which HPV infection is not involved (Cubilla et al. 1996, 1998, 2000, 2004; Barreto et al. 2007; Cunha et al. 2009; Chaux et al. 2010a, 2010b; Chaux and Cubilla 2012; Chaux et al. 2012; Cubilla et al., 2012; Tolstov et al. 2014; Sanchez et al. 2015; Cubilla et al. 2018). According to the WHO 2016 classification system of penile carcinomas, SCC can be classified according to the presence or absence of HPV and by histological findings into several distinct subtypes (Chaux et al. 2010c; Sanchez et al. 2015; Cubilla et al. 2018; Iorga et al. 2020).

Since HPV-related SCCs have a considerably better prognosis than non-HPV-related SCCs, the detection of p16^lnk4a^ (p16), a tumour suppressor protein that is overexpressed in HPV infection, is a reliable human diagnostic test for HPV-related SCCs and is thus also a prognostic factor (Chaux and Cubilla 2012; Sanchez et al. 2015; Dickstein et al. 2016; Moch et al. 2016). In HPV-induced head and neck squamous cell carcinomas, negative expression, or loss, of p16 has been associated with malignant biological behavior and poor prognosis (Albers et al. 2017). Accurate prediction of clinical outcomes in horses is challenging with no recognized prognostic criteria or consistent histopathological classification scheme for eSCC (Romagosa et al. 2011). SCC histopathological subtypes and p16 expression have not been reported in horses, and it is therefore potentially of high clinical importance to provide a quick and reliable method to classify equine SCCs according to their viral or non-viral origin.

Treatment of eSCC is difficult, and a variety of modalities are used with varying degrees of success, and the prognosis can be uncertain (van den Top et al. 2008a, 2008b). Recently, HER-2, a transmembrane tyrosine kinase receptor that, when overexpressed in SCC, produces a continuous growth signal (Silva Amancio et al. 2017), has been explored in human and canine SCC as a negative prognostic factor with promising results (Outh-Gauer et al. 2018). Its detection has enabled new pharmacological treatments utilizing specific monoclonal antibodies (Outh-Gauer et al. 2018). This potential therapeutic target has not been fully investigated in the horse. The aims of this study were to apply a morphological categorization scheme to eSCCs, evaluate p16 and HER-2 expression using IHC, use ISH to screen for the presence of EcPV-2 and test the hypothesis that these factors offer prognostic value.

## 2. Materials and methods

### 2.1. Case selection and clinicopathologic data

The Royal Veterinary College Diagnostic Laboratory and Bell Equine Veterinary Clinic database were reviewed for cases of eSCC affecting the genitalia or ocular/periocular tissues between 2008 and 2024. Where formalin-fixed, paraffin-embedded (FFPE) tissue or haematoxylin and eosin (HE)-stained tissue sections were available, the original histopathological diagnoses of squamous cell carcinoma (SCC), carcinoma *in situ* (CIS), squamous papilloma, or epidermal hyperplasia and dysplasia were reviewed.

The age, sex, neuter status, and breed of each horse, as well as the location of the neoplasm and any relevant clinical features, were obtained. Follow-up data were obtained by electronic questionnaire with the primary practice and by examination of further biopsy submissions from the same cases. The occurrence or absence of tumour-related death was recorded for outcome. Overall survival was recorded for all horses and defined as the time between histologic diagnosis and tumour-related death. Horses that were still alive at the last time of follow-up or died (or were euthanized) due to unrelated causes were censored in the survival analysis. Horses whose records were not obtainable or that were not seen by the practice since the time of diagnosis were not included in the survival data. Any evidence of local recurrence, clinical staging, or metastatic disease was also recorded.

### 2.2. Histopathology

All cases where FFPE tissue blocks or HE-stained tissue sections were available were included for histopathologic examination. HE-stained slides for all the cases were reviewed by a veterinary anatomic pathology resident (KO), two board-certified veterinary anatomic pathologists (ASB, SLP), and one board-certified medical ophthalmic histopathologist (HSM). Histopathologic examination was performed on 4 µm-thick sections stained with HE using light microscopy, confirming the diagnosis of SCC, CIS, squamous papilloma, or epidermal hyperplasia and dysplasia. SCC were evaluated for their predominant histological pattern (Wolff et al. 2013; Cubilla et al. 2018), cellular pleomorphism, mitotic count, presence of intratumoral necrosis or haemorrhage, surface ulceration, and inflammation. Sections were also evaluated for the presence or absence of tumour-free surgeon-cut margins and evidence of vascular or lymphatic invasion.

Cellular pleomorphism was assessed as mild, moderate, or marked. The mitotic count consisted of the number of mitoses in 10 randomly selected, consecutive, non-overlapping (400x) high-power fields (HPF) (equivalent to 2.37 mm^2^) starting at an area of high mitotic activity and avoiding areas of ulceration or necrosis. Intratumoral necrosis was defined as confluent aggregates of necrotic neoplastic cells and recorded as present or absent and excluded areas associated with ulceration. The presence of surface ulceration and inflammation was assessed where possible.

Based on the human WHO 2016 classification system (Cubilla et al. 2018), the following distinct subtypes and morphological variants of penile SCC are currently recognized: SCC (usual, pseudohyperplastic, pseudoglandular), verrucous (pure verrucous, carcinoma cuniculatum), papillary, adenosquamous, sarcomatoid, mixed, basaloid (papillary-basaloid), warty (warty-basaloid, clear cell), lymphoepithelioma-like, and unclassified. For the subset of periocular SCC cases, Yanofsky et al. recognized the following distinct subtypes: carcinoma *in situ* (Bowen's), invasive, clear cell, sarcomatoid, SCC with single-cell infiltrates, *de novo*, verrucous, and lymphoepithelioma-like (Winton et al. 2020). eSCCs (genital or periocular) were classified according to these human systems.

### 2.3. Immunohistochemistry

For immunohistochemistry (IHC), 4 µm-thick paraffin sections were mounted on positively charged slides (SuperFrost Plus; Menzel Gläser, Braunscheig, Germany). Antigen retrieval, labelling, and counterstaining were performed on a Bond-Max Autostainer (Leica Biosystems, Newcastle-upon-Tyne, UK) using the Bond Polymer Refined detection system (Leica Biosystems). The primary antibodies used were against p16 (PA0016, monoclonal [Leica Microsystems, Milton Keynes, UK]; ready-to-use; antigen retrieval in pH 6.0 buffer [ER1, Leica Biosystems] for 10 min) and HER-2 (CB11, monoclonal [Thermo Fisher Scientific, Loughborough, UK]; 1:50 dilution; antigen retrieval in buffer pH 9.0 buffer [ER2, Leica Biosystems] for 20 min). Positive controls included ocular tissue from a human with SCC and haired skin from a canine with SCC (p16) and normal and neoplastic mammary gland tissue from a canine and nonhuman primate (HER-2). The negative controls included the same sections lacking primary antibodies. Isotype controls were used to ensure the specificity of immunolabelling in equine SCCs.

The grading system utilized for assessment of HER-2 expression was previously reported (Wiener et al. 1992), and grades (0, 1+, 2+, and 3+) were assigned based on both the percentage of immunopositive cells and the intensity of labelling. Two systems were utilized for the assessment of p16 expression. One was used in a previous study (Cubilla et al. 2011), and four patterns of p16 expression (0, 1, 2, and 3) were assigned based on the continuity of immunolabelling in neoplastic cells irrespective of the percentage of positive cells, intensity, or localization of immunolabelling. This was supported by the established modified 8th American Joint Committee on Cancer (AJCC) and Union for International Cancer Control (UICC) criteria (Lydiatt et al. 2017) and other human consensus guidelines (Darragh et al. 2012; Singh et al. 2018). We proposed a new grading system modified from a previous study (Cubilla et al. 2011), in which patterns 1 (patchy and discontinuous individual staining in some of the neoplastic cells), 2 (a more extensive albeit discontinuous staining pattern with small clusters of positive neoplastic cells), and 3 (entire and continuous staining in all neoplastic cells), rather than pattern 3 alone, were considered positive.

### 2.4. *In situ* hybridization

A sensitive, multiple gene-targeted *in situ* hybridization (ISH) method was used (RNAscope; Advanced Cell Diagnostics, Hayward, CA), using probes designed to hybridize to EcPV2 E6/E7 DNA or mRNA, to examine a subset of genital SCCs. Using the EcPV2 GenBank reference sequence EU503122.1, 20 probe pairs within a 972-nucleotide region spanning the E6/E7 region were designed and synthesized (EcPV2 E6/E7 (cat. 427111) Advanced Cell Diagnostics). ISH was performed on 5-mm-thick formalin-fixed, paraffin-embedded tissue sections using the RNAscope 2.5 Red assay kit (cat. 322350, Advanced Cell Diagnostics) according to the manufacturer's protocol. Successful hybridization requires the binding of side-by-side probe pairs on the targeted stretch of nucleic acid, initiating a cascade that produces a red reaction product in the presence of the alkaline phosphatase enzyme. The final deposit is red and punctate, with a direct correlation between the amount of the deposit and the number of DNA/RNA target sequences. The controls included both nucleotide-matched scrambled probes and the bacterial probe DapB (cat. 310043 Advanced Cell Diagnostics). A cohort of 29 eSCC were selected for ISH.

Assessment of EcPV-2 was performed semiquantitatively based on the labelling intensity of positive labelling cells, and grades 0, 1+, 2+, and 3+ were assigned. Regardless of the labelling distribution (nuclear vs. cytoplasmic), four grades of EcPV-2 were categorized as follows: 0, complete absence of EcPV-2 labelling in all neoplastic cells; 1, mild labelling intensity in a subset of neoplastic cells; 2, moderate labelling intensity in a subset of neoplastic cells; and 3, strong labelling intensity in a subset of neoplastic cells.

### 2.5. Statistical analysis

Statistical analysis was performed using SPSS Statistics (v. 23; SPSS, Inc., an IBM Company, Chicago, IL). Differences in mitotic count as a numerical variable were assessed using the non-parametric Wilcoxon rank sum test or the Kruskal‒Wallis rank sum test when there were more than two groups per variable. Differences were determined with the chi-square test or Fisher's exact test. Differences were considered significant when *p* < 0.05. For direct comparison of the association of EcPV-2 status with morphological parameters and outcomes, samples were grouped according to their EcPV-2 status derived from ISH. For survival analysis, outcome was defined as the time from histologic diagnosis to death, including due to squamous cell carcinoma-related and unrelated causes or the end of the study period; horses that were lost to follow-up were censored in the survival analysis. The mean survival time (MST) was estimated by the Kaplan‒Meier method and calculated from the period between the date of diagnosis and death or the last known date alive. eSCCs were further divided into six distinct histological subtypes. Descriptive trends were characterized for each parameter between these six groups, as the sample sizes per group were not large enough to warrant statistical analysis.

## 3. Results

### 3.1. Animals and diagnoses

**Figure 1.**
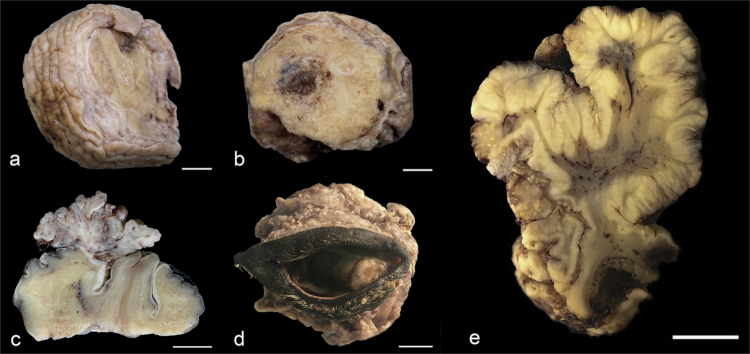
Gross appearance of equine genital and periocular squamous cell carcinoma. (a & b) Penile SCC, basaloid subtype. (c) Penile SCC, verrucous subtype. (d) Ocular SCC, usual/invasive subtype. (e) Preputial SCC, verrucous subtype. Bar = 1 cm.

A total of 122 horses, including a total of 185 samples, were examined in this study. Any one horse may have had one or more benign and/or malignant ocular or genital skin lesions. Of the 122 horses, 123 samples were diagnosed as genital or ocular squamous cell carcinoma (gSCC, oSCC) (Figure 1), 12 as CIS, 23 as papilloma, and 27 as epidermal hyperplasia and/or dysplasia. Follow-up data were obtained from 25 cases.

### 3.2. Clinical characteristics and tumour location

A total of 123 eSCC were identified and reviewed for signalment (age, breed, and castration status), presentation (clinical signs and age of diagnosis), diagnostic (histopathologic biopsy results), treatment, and follow-up data (current survival status, association of euthanasia or natural death with SCC or other disease, and overall survival time post-diagnosis) (Table 1).

**Table 1.** Comparison of clinical, histopathological, immunohistochemical, and *in situ* hybridization features of squamous cell carcinoma in genital and periocular tissue from 123 equine cases, based on histopathological subtype.

	Usual/Invasive (*n* = 91)	Verrucous (*n* = 20)	Warty (*n* = 3)	Basaloid (*n* = 2)	Papillary (*n* = 3)	Pseudoglandular (*n* = 4)
**Patient characteristics**						
**Sex**						
Male	48/91 (53%)	19/20 (95%)	3/3	1/2	2/3	1/4
Female	15/91 (17%)	1/20 (5%)	0/3	1/2	0/3	1/4
Not Specified	28/91 (31%)	0/20 (0%)	0/3	0/2	1/3	2/4
**Age at diagnosis (years)**						
Mean (Range)	14.3 (4–29)	19.3 (11-35)	18	17 (17)	7 (7)	12 (12)
**Breed**						
Cob	23/91 (25%)	2/20 (10%)	0/3	0/2	0/3	1/4
Pony	43/91 (47%)	8/20 (40%)	0/3	1/2	1/3	1/4
Other^a^	48/91 (53%)	12/20 (60%)	3/3	1/2	2/3	3/4
**Tumour location**						
Genital	55/91(60%)	18/20 (90%)	2/3	2/2	2/3	1/4
Periocular	36/91 (40%)	2/20 (10%)	1/3	0/2	1/3	3/4
**Overall survival time (days)^b^**						
Mean (Range)	886 (0–2658)	1554 (609–4836)	522 (409–715)	305 (279–331)	1031 (793–1269)	2974 (1038–4909)
**Recurrence**	15/91 (17%)	5/21 (24%)	0/3	0/2	0/3	0/4
**Diagnosis to local recurrence (days)**						
Mean (Range)	298 (19–1561)	628 (24–1377)	N/A	N/A	N/A	N/A
**Mitotic count (2.37 mm**^2^ **area)**						
Mean (Range)	12.6 (0–66)	16 (4–49)	17 (5–26)	7.5 (4–11)	1.3 (1–2)	3.5 (2–5)
**Necrosis**	39/91 (43%)	9/20 (45%)	2/3	1/2	1/3)	4/4
**Haemorrhage**	12/91 (13%)	1/20 (5%)	1/3	0/2	1/3	0/4
**Vascular Invasion**	6/91 (7%)	3/20 (15%)	0/3	0/2	0/3	0/4
**Lymphatic Invasion**	8/91 (9%)	3/20 (15%)	0/3	0/2	0/3	1/4
**p16**						
* Total positivity*	1/40 (3%)	2/16	0/3	0/2	0/2	0/2
Previous studies (Yanofsky et al. 2011; Wolff et al. 2013; Zhu et al. 2015; Winton et al. 2020)	17/40 (43%)	4/16	2/3	0/2	0/2	2/4
O'Brien et al.	23/40 (58%)	12/16	1/3	2/2	2/2	2/4
Pattern 0	14/40 (35%)	2/16	2/3	0/2	0/2	1/4
Pattern 1	2/40 (5%)	0/16	0/3	0/2	0/2	1/4
Pattern 2	1/40 (3 %)	2/16	0/3	0/2	0/2	0/4
Pattern 3						
**HER-2**						
* Total positivity*	14/39 (36%)	4/16	0/3	1/2	0/2	2/4
0	5/39 (13%)	0/16	0/3	0/2	1/2	1/4
1+	2/39 (5%)	0/16	1/3	0/2	0/2	0/4
2+	18/39(46%)	12/16	2/3	1/2	1/2	1/4
3+	14/39 (36%)	4/16	0/3	½	0/2	2/4
**EcPV-2 status**						
* Total positivity*	5/10	6/8	2/3	2/2	0/2	1/4
0	5/10	2/8	1/3	0/2	2/2	3/4
1+	1/10	2/8	0/3	0/2	0/2	0/4
2+	1/10	3/8	1/3	0/2	0/2	0/4
3+	3/10	1/8	1/3	2/2	0/2	1/4

^a^ ≤ three cases per breed or not provided.

^b^ Days alive at last known assessment. N/A = not applicable. Patterns 0, 1, 2, and 3 for p16 and scores 0, 1+ , 2+ , and 3+ for HER-2 were assigned based on grading schemes utilized in previous papers for p16 (Winton et al. 2020) and HER-2 (Wiener et al. 1992).

The age of the horses at the time of diagnosis ranged between 8 and 35 years, with the age of 73 horses not recorded. Of the 122 total horses, 74 (60.7%) were male or male neutered, 18 (14.8%) were female, and 31 horses (25.4%) were of unspecified sex. The cob and cob cross breeds (26/122; 21.3%) and pony breeds in general (54/122; 44.3%) were the breeds most frequently affected by gSCC and oSCC. The most frequently affected anatomic locations regarding gSCC included the penis not otherwise specified (36/61, 59%), vulva (8/61, 13%), prepuce (8/61, 13%), glans penis (7/61, 12%), and urethral process fossa (2/61, 3%), and those regarding oSCC included the periocular skin (27/62, 44%), eyelid (23/62; 37%), eye (9/62; 15%), cornea (2/62; 3%), and conjunctiva (1/62; 2%).

### 3.3. Histopathology

**Figure 2.**
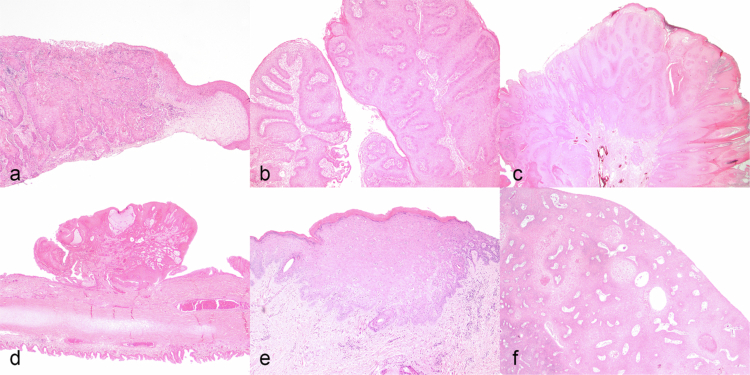
Representative histological types of equine genital and ocular squamous cell carcinoma. (a) Trabeculae and nests of neoplastic epithelial cells with keratin pearls. (b) Exophytic, verrucoid mass with broad-based, straight papillae with rare fibrovascular cores. (c) Exophytic, verrucoid mass with long, undulating papillae with prominent fibrovascular cores. (d) Papillomatous mass with irregular fibrovascular cores and no acanthosis. (e) Infiltrative basophilic neoplastic epithelial cells. (f) Prominent nests with central necrosis and acantholysis. Haematoxylin and eosin.

HE-stained tissue was evaluated in all 185 samples. Of the 123 samples of squamous cell carcinoma, the mitotic count ranged from 0 to 66 (mean = 11.3, standard deviation = 12) per 10 HPF (totalling 2.37 mm^2^). Histologic tissue margins were considered free of neoplastic cells in 91 (74%) samples, with incomplete margins in 32 (26%) samples. In 21 samples (17%), lymphatic or vascular invasion was observed, 15 (71%) of which were in genital tissues and 6 (29%) were within periocular tissues. Intratumoral necrosis was present in 56 of 123 (45.5%) samples. Intratumoral haemorrhage was present in 15 of 123 (12.2%) samples. Of the 123 samples of eSCC, the usual/invasive was the most common histopathological subtype, 91 of 123 (74%), with 20 (16.3%) verrucous, 4 (3.3%) pseudoglandular, 3 (2.4%) papillary, 3 (2.4%) warty, and 2 (1.6%) basaloid (Table 2). The following subtypes and morphological variants were not observed in this study: pseudohyperplastic, lymphoepithelioma-like, clear cell, adenosquamous, sarcomatoid, and mixed. For the subset of oSCC samples, invasive, verrucous subtypes were observed in this study. The characteristic histopathological features of each subtype are outlined in Table 2 and depicted in Figure 2.

**Table 2.** Histopathological classification of equine genital and periocular SCCs.

Subtype	Key histologic features	Frequency (%)
SCC		
Usual/invasive	Keratin pearls	74
Pseudohyperplastic	Prominent hyperkeratosis, flat surface, endophytic nests	0
Pseudoglandular	Nests with central necrosis and acantholysis	3.3
Verrucous		
Pure verrucous	Prominent acanthosis, straight papillae with rare fibrovascular cores, broad-based, no koilocytosis	16.3
Carcinoma cuniculatum	Labyrinthine growth pattern, multifocal areas of dedifferentiation	0
Papillary	Complex papillae with irregular fibrovascular cores, no acanthosis or koilocytosis	2.4
Adenosquamous	Squamous tumour nests with multifocal glandular differentiation	0
Sarcomatoid	Epithelial and spindloid morphology, prominent haemorrhage, and necrosis	0
Mixed	Features of more than one subtype	0
Basaloid	Nests of small-intermediate, basophilic, basaloid cells, many mitoses	1.6
Papillary-basaloid	Features of both papillary and basaloid subtypes	0
Warty	Long, undulating papillae with prominent fibrovascular cores, prominent koilocytosis	2.4
Warty-basaloid	Features of both warty and basaloid subtypes	0
Clear cell	Nests of clear cells with comedonecrosis	0
Lymphoepithelioma-like	Syncytial growth pattern of undifferentiated cells, abundant lymphocytes, plasma cells, and eosinophils obscuring tumour margins	0
Unclassified	No features of the above subtypes	0

Recurrent samples of eSCC were observed only within the usual and verrucous subtypes, with 298–628 days between recurrences. The mitotic counts were highest in the warty and basaloid subtypes and lowest in the papillary subtype. Vascular and lymphatic invasion were most marked in the verrucous subtype, followed by usual/invasive, and absent in the warty, basaloid, and papillary subtypes.

### 3.4. Immunohistochemistry

Immunohistochemical labelling was performed on a subset of 67 eSCC. As per the AJCC and UICC guidelines (Lydiatt et al. 2017) and other established human consensus guidelines (Darragh et al. 2012; Sykora and Brandt 2017), for p16 overexpression, p16 immunolabelling was considered diffuse/block-positive with moderate to strong labelling intensity within the verrucous and usual/invasive subtypes and negative within the remaining subtypes. However, if the proposed modified p16 grading scheme is applied in these horses, p16 immunolabelling is pattern 3 (block-positive) in the warty subtype, patterns 1 and 2 (patchy) in the pseudoglandular, usual, and verrucous subtypes, and pattern 0 (negative) within the basaloid and papillary subtypes. Of those with p16 immunolabelling, pattern 1 was the most common pattern of labelling intensity observed. p16 block-positive labelling was observed in 3/67 samples (5%), with 19/67 (28%) demonstrating patchy weak (pattern 1), 3/67 (5%) patchy moderate (pattern 2), and 3/67 (5%) diffuse moderate to strong (pattern 3) nuclear and/or cytoplasmic labelling. However, if patterns 1 and 2 are also considered block-positive expression of p16, as per the proposed modified grading scheme, then 25 of 67 (37%) SCC samples would be considered p16-block-positive.

**Figure 3.**
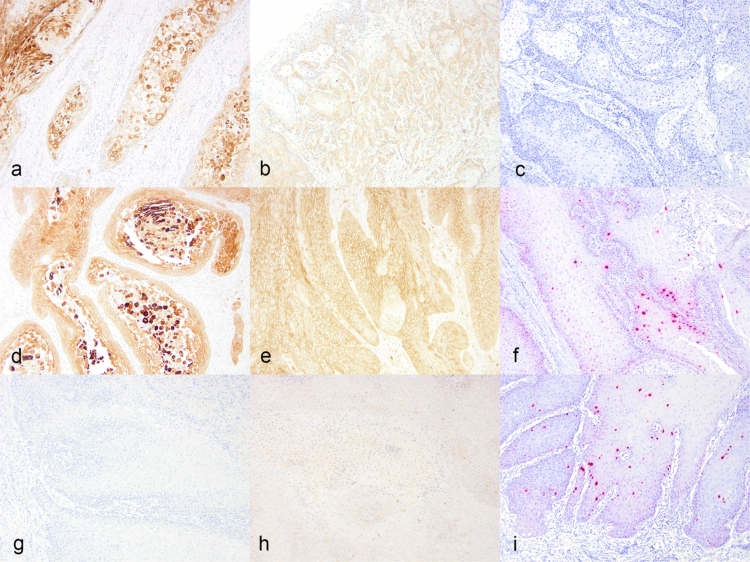
p16, HER-2 and Equus caballus papilloma virus 2 (EcPV-2) detection in equine genital and ocular squamous cell carcinoma. Images (a) to (c) are representative of an ocular usual/invasive subtype. Images (d) to (f) are representative of a verrucous subtype. Images (g) to (i) are representative of a warty subtype. (a) Pattern 1 (p16). (b) Score 1+ (HER-2). (c) Score 0 (EcPV-2 ISH). (d) Pattern 3 (p16). (e) Score 2+ (HER-2). (f) Score 3+ (EcPV-2 ISH). (g) Pattern 1 (p16). (h) Score 1+ (HER-2). (i) Score 2+ (EcPV-2 ISH).

**Figure 4.**
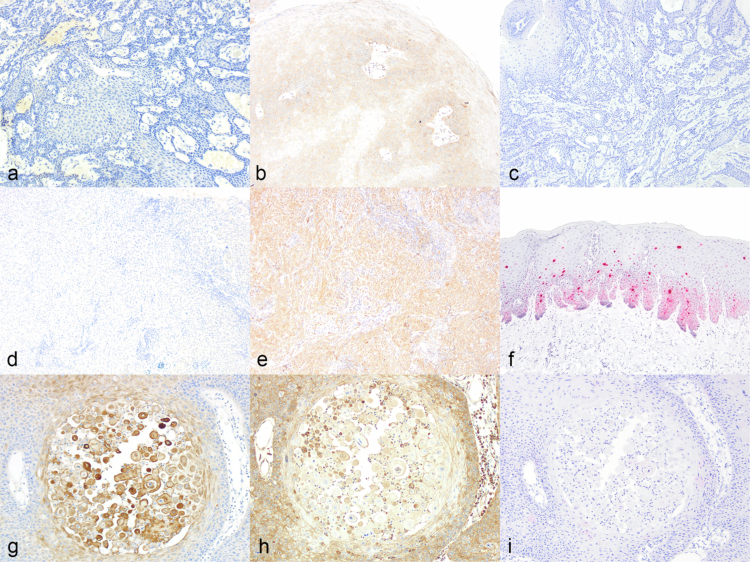
p16, HER-2 and Equus caballus papilloma virus 2 (EcPV-2) detection in equine genital and ocular squamous cell carcinoma. Images (a) to (c) are representative of a papillary subtype. Images (d) to (f) are representative of a basaloid subtype. Images (g) to (i) are representative of a pseudoglandular subtype. (a) Pattern 0 (p16). (b) Score 0 (HER-2) (c) Score 0 (EcPV-2 ISH). (d) Pattern 0 (p16). (e) Score 2+ (HER-2) (f) Score 3+ (EcPV-2 ISH). (g) Pattern 2 (p16). (h) Score 3+ (HER-2). (i) Score 0 (EcPV-2 ISH).

HER-2 immunolabelling was observed in the usual/invasive, verrucous, basaloid, and pseudoglandular subtypes and absent within the warty and papillary SCC subtypes. Although a score of only 3+ is considered positive, as per human SCC guidelines (Wolff et al. 2013), scores of 2+ and 3+ are the most common labelling intensities observed. HER-2 positivity was widely observed (21/66; 32%), with 3/66 (5%) demonstrating weak, 35 (53%) moderate, and 21 (32%) strong membranous and/or cytoplasmic labelling. HER-2 was also considered positive in the majority of the examined (18/27; 67%) SCC precursor samples, which included five CIS samples, nine papilloma samples, and 13 epidermal hyperplasia or dysplasia samples. However, if scores of 1+ and 2+ are also considered positive, then 59 of 66 (89%) SCC samples and 27 of 27 (100%) SCC precursor samples would be considered HER-2 positive. Representative images are depicted in Figures 3 and 4.

### 3.5. *In situ* hybridization

*In situ* hybridization was performed on 29 representative samples of six histopathological subtypes (usual/invasive, verrucous, papillary, warty, basaloid, and pseudoglandular) (Figures 3, 4 and Table 1). These included both 19 gSCC and 10 oSCC. Robust E6/E7 probe hybridization was detected in 16/29 (55%) samples (15gSCC, 1oSCC), with 3/16 demonstrating weak (1+) labelling, 5/16 moderate (2+) labelling, and 8/16 strong (3+) labelling. Positive labelling was observed within the suprabasal layers (3/16) or both the suprabasal and basal layers (13/16%) of the neoplastic stratified squamous to columnar epithelium in areas of both neoplastic and hyperplastic proliferation. EcPV-2 probe hybridization ranged from diffuse nuclear within the suprabasal layers to granular cytoplasmic within the basal layer in all 16 samples. EcPV-2 positivity was observed in all the examined subtypes, apart from the papillary subtype. A score of 3+ was the most common labelling intensity observed. There was no association between EcPV-2 status and labelling intensity for HER-2 or P16 expression or mitotic count.

### 3.6. Clinical outcome and survival analysis

**Figure 5.**
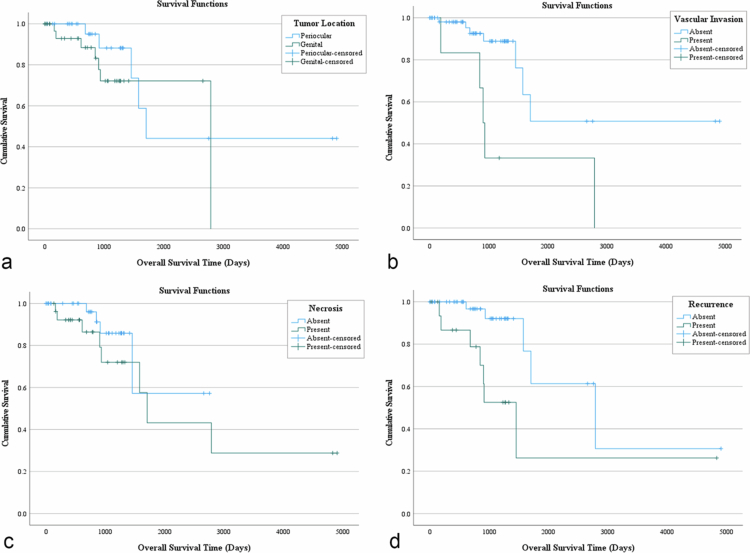
Kaplan‒Meier survival analysis for equine squamous cell carcinoma based on overall squamous cell carcinoma population. (a) Tumour location, (b) vascular invasion, (c) necrosis, (d) and recurrence.

Follow-up data, including date of death, were available for 21 of the 122 horses (17.2%) in which histopathologic evaluation was performed (Figure 5). The survival of all horses ranged from 0 days to 4926 days, with a median survival time (MST) of 1704 days. The MST of horses with oSCC was 1704 days, and 2789 days for horses with gSCC (*n* = 61). If vascular invasion was present, the MST was 904 days compared to 1704 if absent. The MST for horses with intratumoral necrosis was 1704 days compared to 2757 if absent. The MST for horses with p16-negative immunolabelling, according to our proposed modified p16 grading system, was 793 days, compared to 1115 days if p16-positive. Of the 21 horses with follow-up data, 11 (52%) were euthanized or died of causes perceived to be related to their epithelial neoplasia. In horses with recurrence, the MST was 1453 days compared to 2789 days in horses without recurrence. The mean survival time was highest in the pseudoglandular subtype, then verrucose, and lowest in the basaloid subtype. Clear surgical margins were present in 91 of 123 (74%) samples of eSCC. Local recurrence of SCC was recorded in 20 samples (16.3%).

Intratumoral necrosis (*p* = 0.05), vascular invasion (*p* < 0.001), and tumour recurrence (*p* = 0.003) were identified to be independent predictors of survival. If the modified p16 grading scheme was applied to this sample population, p16-negative immunolabelling would be considered an additional independent predictor of survival (*p* = 0.02). Sex, breed, age at diagnosis, tumour localization, mitotic count, haemorrhage, presence of lymphatic invasion, HER-2 status, and EcPV-2 status were not significantly associated with survival. The p16 and HER-2 labelling intensity and distribution were not associated with survival.

## 4. Discussion

In this retrospective equine squamous cell carcinoma study, we aimed to histologically subtype a large case series of eSCCs and correlate with p16 and HER-2 expression, EcPV-2 status, and various clinical and histopathological parameters to predict tumour behaviour and prognosis.

Using the WHO 2016 classification for humans with squamous cell carcinoma, cases were divided into six subtypes based on the predominant histological features of the tumour. All the observed histological subtypes of squamous cell carcinoma (usual/invasive, verrucous, warty, basaloid, papillary, and pseudoglandular) were recorded. The usual/invasive carcinoma was the most common subtype, followed by verrucous, which is consistent with previous studies in humans (Chaux and Cubilla 2012). All the observed eSCC subtypes occurred more commonly in genital tissue than in periocular tissue, apart from the pseudoglandular subtype. This may be related to the different types of lining epithelium for these tissues, which are often keratinized stratified squamous for penile samples and nonkeratinized stratified columnar to squamous for periocular samples. This is the first time equine SCCs have been histologically subtyped according to the WHO scheme. A previous publication investigated a cohort of equine penile lesions that included hyperplastic lesions and CIS and SCC cases (Ramsuer et al. 2019). The authors also identified by ISH, particular signal distribution patterns allowing the differentiation of early (hyperplasia, papilloma) from late-stage penile epithelial lesions (Ramsuer et al. 2019). Here, we present histopathological differences between invasive subtypes of eSCC, which are similar to those described by the WHO. Further studies comparing these proposed histopathological classifications are needed to validate their clinical significance.

Recurrent cases of squamous cell carcinoma were only observed within the usual and verrucous subtypes, with about 298–628 days between recurrences. These findings suggest that these two subtypes are more common and more biologically aggressive than the remaining examined subtypes. However, this may also reflect the considerably larger number of samples in these two subtypes compared to the other four, which were less frequently observed. The mitotic count was highest in the warty and basaloid subtypes and lowest in the papillary subtype. Owing to the small sample sizes for warty, basaloid, papillary, and pseudoglandular subtypes, statistical analysis between the subgroups was not feasible. Therefore, the descriptive trends characterized for this cohort may not be representative of the described subtypes. Additional cases of eSCC are needed for a better understanding of their biological behavior and prognosis.

To understand the molecular pathways of eSCC, the expression of a cell cycle regulator (p16) and cellular growth factor (HER-2) was investigated in a representative proportion of cases. p16^INK4a^ (p16) is a tumour suppressor protein that regulates the cell cycle at the G1/S checkpoint via the inhibition of cyclin D-CDK4/6. Its role in tumour progression and prognosis has been studied in various human tumours (Romagosa et al. 2011; Albers et al. 2017; Farzanehpour et al. 2020); however, few studies have investigated p16 expression in horses.

Given that there are few data regarding p16 immunohistochemistry in equine tissue and that we do not yet know what p16 value is considered significant in equine SCC cases, two scoring systems have been applied to these cases. The first scoring system applied in human anogenital SCC grading, based on four patterns of p16 expression, which state that positive p16 is based on greater than or equal to 75% labelling of neoplastic epithelial cells with moderate labelling intensity (Cubilla et al. 2011; Lydiatt et al. 2017). The second scoring system utilized in this study was a modified version of a published method for human SCC (Cubilla et al. 2011), so that SCC samples with p16 labelling patterns 1 through 3 were considered positive rather than only pattern 3.

In the equine SCCs examined in this study, the expression of p16 was common to four of the six examined subtypes, with the strongest expression in the warty, pseudoglandular, and usual/invasive subtypes. However, if analysis is performed following methods established for humans (Darragh et al. 2012; Lydiatt et al. 2017; Silva Amancio et al. 2017), p16 overexpression was observed only within the usual/invasive and verrucous subtypes. p16 expression in select subtypes, according to the modified grading system, is similar to previous studies in human SCCs (Albers et al. 2017) and thus appears that it is not fundamental for neoplasia development in equine SCCs. Of those cases with p16 positivity, pattern 1 was the most common labelling pattern observed.

HER-2 is a tyrosine kinase receptor that, when overexpressed in cases of SCC due to gene amplification, produces a continuous growth signal (Scott et al. 2003) that can result in neoplasia. In this study, HER-2 was present in most SCCs and pre-neoplastic lesions, although it was lowest in papillary carcinomas, thus identifying this receptor as a potential therapeutic target.

EcPV-2 positivity was observed in all examined subtypes, apart from the papillary subtype, and was particularly high in the basaloid, verrucous, and warty subtypes. This is similar to previous human studies (Kurman et al. 1993; Gregoire et al. 1995; Gross and Pfister 2004; Chaux and Cubilla 2012; Cubilla et al. 2018), which demonstrated a strong association between HPV positivity and the warty, basaloid, and warty-basaloid subtypes and consistent negativity in papillary carcinomas. However, in these studies, the verrucous subtype is consistently HPV-negative, whereas in this study, 75% of the verrucous were EcPV-2 positive. These differences in EcPV-2 status between the various observed eSCC subtypes and those established in various human studies may be related to the small sample sizes in most of the equine SCC subtype groups. Additional cases for each eSCC subtype are needed for confirmation. Only 1 out of 10 oSCC was EcPV-2 positive, which is in accordance with a recent study (Tuomisto et al. 2024).

In samples with positive EcPV-2 status, EcPV-2 probe hybridization within the neoplastic stratified squamous epithelium ranged from diffuse nuclear within the suprabasal layers to granular cytoplasmic within the basal layer in all 16 samples. These results are consistent with those of a previous study, which concluded that stronger probe hybridization within the suprabasal layers is reflective of higher viral copy numbers (Yanofsky et al. 2011).

Given the link between the presence of HPV and squamous cell carcinoma in humans, we aimed to assess the presence and relationship of EcPV-2 in equine squamous cell carcinomas. In previous studies (Bogaert et al. 2012; Kainzbauer et al. 2012; Knight et al. 2013; Lange et al. 2013; Fischer et al. 2014; Sanchez et al. 2015; Sykora et al. 2017; Ramsuer et al. 2019; Tuomisto et al. 2024), EcPV-2 has been reported in both neoplastic and non-neoplastic genital and ocular lesions. Complementing a previous study using ISH and reverse transcriptase PCR (Wolff et al. 2013), oncogenic E6 and E7 transcripts of EcPV-2 were detected by ISH in genital and periocular eSCCs, which suggests an active pathogenic role of the virus. The labelling intensity and distribution of p16 and HER-2 expression were not associated with EcPV-2 status.

p16 immunohistochemistry has previously been evaluated for its potential to act as a proxy for HPV detection via PCR (Cubilla et al. 2011). This is due to the upregulation of p16 secondary to the expression of the E7 oncogene of HPV (Cubilla et al. 2011; Albers et al. 2017; Iorga et al. 2020). In contrast, there was no association between p16 positivity and the presence of EcPV-2 in this study, suggesting that p16 immunohistochemistry may not serve as a surrogate for EcPV-2 testing by ISH. Further studies comparing the E7 gene and protein structure between human and equine papillomaviruses are needed.

In eSCCs, the specific parameters associated with decreased overall survival include intratumoral necrosis, vascular invasion, and tumour recurrence. Vascular and lymphatic invasion were highest in the verrucous subtype, then usual/invasive, and were absent in the warty, basaloid, and papillary subtypes. Vascular invasion was similarly associated with reduced survival in reported cases of human SCC (Chaux and Cubilla 2012). It is often observed in more advanced cases and has been identified as a significant predictor of metastasis (Chaux and Cubilla 2012). Although survival analysis was limited for the subgroups, the survival time was highest in the pseudoglandular subtype, then warty, and lowest in the basaloid subtype. In contrast to previous human and canine studies identifying HER-2 expression in SCCs as a negative prognostic factor (Outh-Gauer et al. 2018), HER-2 expression was not significantly associated with overall survival in this study.

## 5. Conclusions

For the first time, six histological subtypes of equine squamous cell carcinoma were identified in accordance with WHO guidelines, with the usual/invasive type being most frequent. Intratumoral necrosis, vascular invasion, and tumour recurrence were all associated with shorter survival times in equine genital and periocular SCC cases. HER-2 and EcPV-2 status were not associated with survival time. Although possible for human HPV-induced squamous cell carcinomas, p16-block positivity appears not to be a reliable indicator of EcPV-2 status in horses. Further studies focusing on p16 expression in equine SCC are necessary. HER-2 over-expression occurs in SCC and precursor lesions, identifying this receptor as a potential therapeutic target. This study contributes further evidence supporting the etiological role of EcPV-2 in equine gSCC and its association with a subset of oSCC. The histopathological subtyping system, histologic criteria, and immunohistochemical assay described here could be easily transferred to diagnostic pathology laboratories for maximum benefit to the equine population.
